# What is a placental mammal anyway?

**DOI:** 10.7554/eLife.30994

**Published:** 2017-09-12

**Authors:** Patrick Abbot, John A Capra

**Affiliations:** Department of Biological SciencesVanderbilt UniversityNashvilleUnited States

**Keywords:** Tammar wallaby, placenta, lactation, transcriptomics, reproduction, marsupial, Other

## Abstract

Many developmental functions in marsupials and eutherian mammals are accomplished by different tissues, but similar genes.

**Related research article** Guernsey MW, Chuong EB, Cornelis G, Renfree MB, Baker JC. 2017. Molecular conservation of marsupial and eutherian placentation and lactation. *eLife* **6**:e27450. doi: 10.7554/eLife.27450

Most of us learned in school that there are three kinds of living mammals — eutherians, marsupials and monotremes — and that the most obvious differences between them are how they reproduce. The eutherian or 'placental' mammals, like humans, make up the vast majority of today's mammalian diversity. Eutherians all have a chorioallantoic placenta, a remarkable organ that forms after conception at the site where the embryo makes contact with the lining of the mother's uterus (Langer, 2008).

Marsupials and monotremes handle pregnancy differently (Abbot and Rokas, 2017; Renfree, 2010). Egg-laying monotremes, like the duck-billed platypus, have tiny 'puggles' that hatch from leathery shells. Marsupials — the kangaroos, koalas, bandicoots, opossums and so on — have live births, but their pregnancies are brief and their tiny joeys are developmentally immature, and would seem to have little need of a placenta. After birth, the joeys continue to develop outside of their mother's body, often within folds and pouches on their mother's abdomen.

In marsupials, the milk provided by the mother after birth is central to the development of the offspring and, unlike in eutherian mammals, the composition of this milk changes dramatically as the young joeys grow. In essence, the mammary glands of marsupials perform many of the functions of the eutherian placenta (Renfree, 2010; Sharp et al., 2017; Figure 1). And to upend what you may have learned in biology class even more, marsupials do have a placenta after all, but it develops late in pregnancy and from different tissues compared with eutherians. Thus, the difference between eutherian mammals and marsupials is not the presence or absence of a placenta, but rather the relative emphasis put on placentation and lactation to nurture offspring through development.

**Figure 1.**
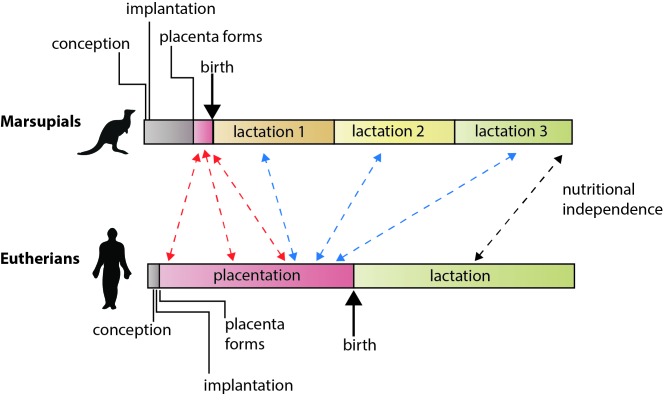
The different reproductive strategies of eutherian mammals and marsupial mammals. In eutherians, the energy invested by the mother in rearing young before birth (via placentation) and after birth (via lactation) is roughly equally. In marsupials, gestation is brief, the placenta forms late in pregnancy, and lactation is extended. Guernsey et al. show that genetic features that regulate development via the placenta in eutherians are shared with the short-lived marsupial placenta (red arrows). They also show that some of the genes that underlie placental functions in eutherians are expressed during lactation in marsupials (blue arrows), including various conserved components of lactation itself (black arrow; Lefèvre et al., 2010). Note: time scales are not absolute.

Now, in eLife, Julie Baker of Stanford University School of Medicine, Marilyn Renfree of the University of Melbourne and co-workers — including Michael Guernsey of Stanford as first author, Edward Chuong of the University of Utah and Guillaume Cornelis (Stanford) — report new details of the molecular mechanisms underlying placentation and lactation in eutherians and marsupials (Guernsey et al., 2017). The results were obtained by using a modified version of a technique called RNA-seq to measure how the transcriptome (the complete set of RNA transcripts in a cell or set of cells) varied between different cells types during development (Rokas and Abbot, 2009).

Guernsey et al. compared changes in gene expression in two cell types in the placenta of a tammar wallaby, a small Australian marsupial, during development. They found that gene expression differed between the two tissues and, moreover, that it changed dynamically over time, similar to what happens in eutherians. Furthermore, among the transcripts they found many that had critical functions in eutherian placentas, including members of the *Ig7* signaling pathways and *GCM1*, a transcription factor that is important in the formation and development of the placenta. And it was not simply the genes that were conserved, the patterns of gene expression in the wallaby placenta resembled those seen in the mouse placenta in the early stages of pregnancy. This is exactly what we would expect to see if the placenta performs early developmental functions in the wallaby, with later functions being provided post-natally. This suggests that an essential difference between marsupials and eutherians is not in the early functions of the placenta, but rather in how placental functions have been compartmentalized over the course of the evolution of eutherian pregnancy.

Finally, Guernsey et al. characterized the patterns of gene expression in the mammary glands of the tammar and several mammals. Both mouse and wallaby shared similar patterns of gene expression, underscoring the theme of functional compartmentalization and conservation in both groups. But most remarkably, they identified a number of genes expressed in the mammary glands in the tammar that are known to be functionally important in the placenta in eutherians (Figure 1). These genes included genes involved in nutrient transport and several known to be required for eutherian placentation (including *GCM1*). This conservation of gene expression argues that in marsupials the placenta manages early fetal development and lactation manages late fetal development, using some of the same genes and molecular pathways as the eutherian placenta.

Those who study marsupials have long argued that we need to correct our textbooks to acknowledge marsupisal placentas and their distinctively complex lactation (Renfree, 1983). Guernsey et al. strengthen the case by demonstrating that both eutherians and marsupials express a conserved toolkit of genes that may be localized to different tissues and organs, but serve common purposes in fetal development. This surprising conservation underscores the importance of identifying the genes underlying functional changes during evolution (Rausher and Delph, 2015).

Looking ahead, it is worth noting that marsupials vary tremendously in reproductive traits (Tyndale-Briscoe, 2005), and that characterizing more species in the way that Guernsey et al. have done for the tammar wallaby will provide a richer understanding of the evolution and diversity of marsupial pregnancy itself. However, more work is needed to develop appropriate statistical methods for quantifying the conservation of transcriptome profiles between species. And looking beyond mammals, forms of placentation are found in everything from lizards, to seahorses, to insects, and preliminary studies indicate that many of the genes or traits involved are shared (Ostrovsky et al., 2016; Whittington et al., 2015). It will be fascinating to learn how deeply we can trace the origins of the pregnancy toolkit.

## References

[bib1] Abbot P, Rokas A (2017). Mammalian pregnancy. Current Biology.

[bib2] Guernsey MW, Chuong EB, Cornelis G, Renfree MB, Baker JC (2017). Molecular conservation of marsupial and eutherian placentation and lactation. eLife.

[bib3] Langer P (2008). The phases of maternal investment in eutherian mammals. Zoology.

[bib4] Lefèvre CM, Sharp JA, Nicholas KR (2010). Evolution of lactation: ancient origin and extreme adaptations of the lactation system. Annual Review of Genomics and Human Genetics.

[bib5] Ostrovsky AN, Lidgard S, Gordon DP, Schwaha T, Genikhovich G, Ereskovsky AV (2016). Matrotrophy and placentation in invertebrates: a new paradigm. Biological Reviews.

[bib6] Rausher MD, Delph LF (2015). When does understanding phenotypic evolution require identification of the underlying genes?. Evolution.

[bib7] Renfree MB, Finn C. A (1983). Marsupial reproduction: the choice between placentation and lactation. Oxford Reviews of Reproductive Biology.

[bib8] Renfree MB (2010). Marsupials: placental mammals with a difference. Placenta.

[bib9] Rokas A, Abbot P (2009). Harnessing genomics for evolutionary insights. Trends in Ecology & Evolution.

[bib10] Sharp JA, Wanyonyi S, Modepalli V, Watt A, Kuruppath S, Hinds LA, Kumar A, Abud HE, Lefevre C, Nicholas KR (2017). The tammar wallaby: a marsupial model to examine the timed delivery and role of bioactives in milk. General and Comparative Endocrinology.

[bib11] Tyndale-Briscoe H (2005). Life of Marsupials.

[bib12] Whittington CM, Griffith OW, Qi W, Thompson MB, Wilson AB (2015). Seahorse brood pouch transcriptome reveals common genes associated with vertebrate pregnancy. Molecular Biology and Evolution.

